# Supplementary material for: Multi‐dataset identification of innovative feature genes and molecular mechanisms in keratoconus
Source: J Cell Mol Med. 2024 Sep 19;28(18):e70079. doi: 10.1111/jcmm.70079 (PMC11412914; doi:10.1111/jcmm.70079)
Supplement: Supplementary file 2 — Material S2. [file JCMM-28-e70079-s002.zip › 7.GSEA analysis/5.RNF217-AS1/KEGG_FC_EPSILON_RI_SIGNALING_PATHWAY.html]

Details for gene set KEGG\_FC\_EPSILON\_RI\_SIGNALING\_PATHWAY[GSEA]

|  || Dataset | RNF217-AS1.group.cls#h\_versus\_l |
| Phenotype | group.cls#h\_versus\_l |
| Upregulated in class | l |
| GeneSet | KEGG\_FC\_EPSILON\_RI\_SIGNALING\_PATHWAY |
| Enrichment Score (ES) | -0.38812625 |
| Normalized Enrichment Score (NES) | -1.3778124 |
| Nominal p-value | 0.061601643 |
| FDR q-value | 0.3753526 |
| FWER p-Value | 0.888 |
Table: GSEA Results Summary

  

Fig 1: Enrichment plot: KEGG\_FC\_EPSILON\_RI\_SIGNALING\_PATHWAY      
 Profile of the Running ES Score & Positions of GeneSet Members on the Rank Ordered List

  

| SYMBOL | TITLE | RANK IN GENE LIST | RANK METRIC SCORE | RUNNING ES | CORE ENRICHMENT || 1 | PIK3R5 | na | 1058 | 0.434 | 0.0157 | No |
| 2 | IL3 | na | 4732 | 0.226 | -0.0663 | No |
| 3 | PLA2G4E | na | 4967 | 0.218 | -0.0498 | No |
| 4 | PLA2G2F | na | 5587 | 0.196 | -0.0469 | No |
| 5 | CSF2 | na | 6383 | 0.171 | -0.0516 | No |
| 6 | PIK3CD | na | 6778 | 0.159 | -0.0460 | No |
| 7 | RAC3 | na | 7163 | 0.148 | -0.0413 | No |
| 8 | PLA2G2C | na | 7467 | 0.141 | -0.0351 | No |
| 9 | PLA2G2D | na | 7735 | 0.134 | -0.0285 | No |
| 10 | IL13 | na | 8644 | 0.113 | -0.0427 | No |
| 11 | MAPK12 | na | 9095 | 0.103 | -0.0447 | No |
| 12 | VAV2 | na | 9125 | 0.103 | -0.0346 | No |
| 13 | SOS1 | na | 9467 | 0.096 | -0.0343 | No |
| 14 | PLA2G12B | na | 10412 | 0.079 | -0.0531 | No |
| 15 | JMJD7-PLA2G4B | na | 10713 | 0.074 | -0.0538 | No |
| 16 | MAPK13 | na | 10953 | 0.071 | -0.0532 | No |
| 17 | PIK3CB | na | 11439 | 0.064 | -0.0604 | No |
| 18 | KRAS | na | 11445 | 0.064 | -0.0538 | No |
| 19 | MAPK11 | na | 12121 | 0.054 | -0.0675 | No |
| 20 | PDPK1 | na | 12622 | 0.047 | -0.0770 | No |
| 21 | MAPK9 | na | 12895 | 0.044 | -0.0802 | No |
| 22 | RAC2 | na | 12945 | 0.043 | -0.0770 | No |
| 23 | MAPK1 | na | 13362 | 0.038 | -0.0850 | No |
| 24 | TNF | na | 13583 | 0.035 | -0.0876 | No |
| 25 | PLCG1 | na | 13642 | 0.035 | -0.0856 | No |
| 26 | PIK3CA | na | 14021 | 0.031 | -0.0933 | No |
| 27 | MAPK3 | na | 14127 | 0.029 | -0.0932 | No |
| 28 | PLA2G10 | na | 14207 | 0.028 | -0.0925 | No |
| 29 | MAPK14 | na | 14311 | 0.027 | -0.0926 | No |
| 30 | MAP2K7 | na | 14547 | 0.024 | -0.0968 | No |
| 31 | PLA2G2E | na | 14946 | 0.020 | -0.1062 | No |
| 32 | PRKCD | na | 15568 | 0.013 | -0.1227 | No |
| 33 | MAPK10 | na | 15620 | 0.013 | -0.1228 | No |
| 34 | AKT2 | na | 15816 | 0.011 | -0.1273 | No |
| 35 | HRAS | na | 15878 | 0.010 | -0.1280 | No |
| 36 | PIK3R1 | na | 16145 | 0.007 | -0.1349 | No |
| 37 | MAP2K2 | na | 16236 | 0.006 | -0.1368 | No |
| 38 | MAP2K4 | na | 16303 | 0.006 | -0.1381 | No |
| 39 | PLA2G6 | na | 16529 | 0.003 | -0.1442 | No |
| 40 | MAPK8 | na | 16681 | 0.002 | -0.1484 | No |
| 41 | PLA2G4A | na | 17031 | -0.002 | -0.1583 | No |
| 42 | LAT | na | 17047 | -0.002 | -0.1585 | No |
| 43 | MAP2K3 | na | 17092 | -0.003 | -0.1595 | No |
| 44 | FYN | na | 17143 | -0.003 | -0.1606 | No |
| 45 | NRAS | na | 17304 | -0.005 | -0.1647 | No |
| 46 | AKT1 | na | 18280 | -0.013 | -0.1914 | No |
| 47 | RAC1 | na | 18357 | -0.014 | -0.1921 | No |
| 48 | INPP5D | na | 18494 | -0.015 | -0.1944 | No |
| 49 | BTK | na | 18772 | -0.018 | -0.2005 | No |
| 50 | RAF1 | na | 18969 | -0.020 | -0.2041 | No |
| 51 | SOS2 | na | 19710 | -0.027 | -0.2225 | No |
| 52 | PLA2G12A | na | 20757 | -0.037 | -0.2487 | No |
| 53 | GRB2 | na | 22374 | -0.055 | -0.2896 | No |
| 54 | PIK3R2 | na | 22894 | -0.061 | -0.2981 | No |
| 55 | MAP2K1 | na | 23215 | -0.064 | -0.3005 | No |
| 56 | IL5 | na | 23582 | -0.070 | -0.3037 | No |
| 57 | PRKCA | na | 23805 | -0.072 | -0.3024 | No |
| 58 | SYK | na | 24484 | -0.082 | -0.3132 | No |
| 59 | VAV3 | na | 24686 | -0.086 | -0.3099 | No |
| 60 | PLCG2 | na | 24806 | -0.087 | -0.3040 | No |
| 61 | LYN | na | 24892 | -0.089 | -0.2970 | No |
| 62 | FCER1G | na | 27239 | -0.131 | -0.3508 | No |
| 63 | LCP2 | na | 28164 | -0.153 | -0.3612 | No |
| 64 | GAB2 | na | 28273 | -0.155 | -0.3478 | No |
| 65 | PLA2G1B | na | 29143 | -0.180 | -0.3538 | No |
| 66 | PIK3CG | na | 30335 | -0.218 | -0.3650 | Yes |
| 67 | PLA2G5 | na | 30416 | -0.220 | -0.3439 | Yes |
| 68 | MS4A2 | na | 31176 | -0.249 | -0.3393 | Yes |
| 69 | PRKCE | na | 31530 | -0.265 | -0.3213 | Yes |
| 70 | AKT3 | na | 31832 | -0.281 | -0.3000 | Yes |
| 71 | MAP2K6 | na | 32259 | -0.307 | -0.2797 | Yes |
| 72 | PIK3R3 | na | 32895 | -0.352 | -0.2605 | Yes |
| 73 | PLA2G2A | na | 33013 | -0.361 | -0.2255 | Yes |
| 74 | IL4 | na | 33228 | -0.378 | -0.1915 | Yes |
| 75 | PLA2G3 | na | 33574 | -0.415 | -0.1573 | Yes |
| 76 | VAV1 | na | 34083 | -0.499 | -0.1189 | Yes |
| 77 | PRKCB | na | 34175 | -0.520 | -0.0663 | Yes |
| 78 | FCER1A | na | 34631 | -0.773 | 0.0028 | Yes |
Table: GSEA details [plain text format]

  

Fig 2: KEGG\_FC\_EPSILON\_RI\_SIGNALING\_PATHWAY      
 Blue-Pink O' Gram in the Space of the Analyzed GeneSet

  

Fig 3: KEGG\_FC\_EPSILON\_RI\_SIGNALING\_PATHWAY: Random ES distribution      
 Gene set null distribution of ES for **KEGG\_FC\_EPSILON\_RI\_SIGNALING\_PATHWAY**
